# Inclusion of the homeless in health equity curricula: a needs assessment study

**DOI:** 10.1080/10872981.2020.1777061

**Published:** 2020-06-23

**Authors:** Corinne T. Feldman, Gregory D. Stevens, Enya Lowe, Desiree A. Lie

**Affiliations:** Keck School of Medicine, University of Southern California, Alhambra, CA, USA

**Keywords:** Needs assessment, vulnerable patients, homelessness, attitudes, mixed method

## Abstract

Exposure to homeless patients is a potential strategy to teach about social determinants of health and health inequities. Little is known about student attitudes and preferences for learning about the homeless in curricula addressing vulnerable populations. A needs assessment to determine student readiness may inform strategies for teaching. A mixed-methods study of one matriculating physician assistant student class, with a cross-sectional survey and 3 focus groups (FG). The validated 19-item Health Professionals’ Attitudes Toward Homelessness inventory (HPATHI) and new 7-item Learning Attitudes scale were administered to explore perceptions and preferences about relevance of caring for the homeless to future practice. FGs were conducted to theme saturation. Verbatim transcripts were independently read and coded by 3 researchers using constant comparison. Survey response rate was 100% (N = 60). Overall HPATHI mean score was 3.97 ± 0.04 of 5, indicating positive attitudes toward the homeless. The highest mean score (4.26 ± 0.04) was for the social advocacy subscale; the lowest (3.02 ± 0.06) for personal advocacy. The Learning Attitude scale (Cronbach’s alpha 0.89) mean score was 4.47 ± 0.07 out of 5, showing a positive attitude toward curricular exposure. Older students and those with prior experience with the homeless had higher HPATHI scores (p < 0.05). Four major themes emerged: vulnerable patients cannot advocate for themselves; learning about homelessness is relevant to future practice; preference for multiple teaching strategies and adequate preparation for street rotations; and anticipated anxiety about safety. Students recognize the value of learning from homeless patients as part of gaining skills in caring for vulnerable populations. Experiential learning opportunities focusing on this group are seen as an acceptable and valuable way to gain skills applicable to all vulnerable patients. Students express fear and anxiety around non-traditional settings such as the street. Their anxieties should be adequately addressed when designing clinical rotations.

## Introduction

Current accreditation standards of most health professions training programs recommend teaching that incorporates social determinants of health and vulnerable populations to address health disparities that will confront all future providers [1–4]. While educators have focused on exposing their trainees to a variety of vulnerable populations including immigrants, the poor, non-English-speaking and ethnic minorities, less attention has been paid to other marginalized groups such as the homeless and the incarcerated. In 2018, over a half million of 330 million individuals in the USA (US) experienced homelessness on any given night with 35% sleeping unsheltered [5]. The homeless have been shown to suffer increased morbidity and mortality and multiple, poor social determinants of health in comparison to their housed counterparts [6]. As homelessness becomes a common social condition within large urban and some rural settings [5,7,8], medical educators have sought to include care of the homeless within their curricula to address health disparities [9,10]. Yet, little is known about student attitudes toward curricula inclusive of caring for the homeless as a vulnerable population, particularly in a street setting. Street Medicine [11] is the delivery of healthcare in a setting most acceptable to unsheltered patients, such as on a park bench, sidewalk or under a bridge. Providing care ‘on the streets’ allows access to health care that is not traditionally available to the homeless, who trainees typically see in emergency rooms or urgent care settings. Intentional exposure to street medicine has the potential to provide in-depth teaching about addressing disparities, compared with passive exposure rotations in provider-centered ambulatory or hospital settings [12–15].

The goal of this study was to explore student attitudes toward people experiencing homelessness and a curriculum inclusive of Street Medicine, as a needs assessment to design a longitudinal equity-based curriculum to address health disparities. Because health professions students tend to respond in a socially desirable manner to surveys [16–18] we included a qualitative component to examine the reasoning and emotions associated with their attitudes and preferences. We hypothesized first, that matriculating students would have positive attitudes toward supporting and working with the homeless; and second, that they would express both positive reactions and concerns about rotations involving homeless patients.

## Methods

### Study setting

The study was conducted at a 33-month Master of Physician Assistant (PA) Practice program in Los Angeles, California, graduating approximately 60 students a year, on the matriculating class graduating in 2022. Los Angeles is the second most populous metropolitan area in the US after New York City and ranks 9th in the Global Economic Power Index. It is home to over 18 million and has the highest number of homeless persons (53,000 at last count) with 3 out of 4 homeless persons remaining unsheltered in 2019 [5,19].

### Study design

This mixed-methods study involved a cross-sectional survey and three focus groups (FGs). The survey examined student attitudes towards working with people who experience homelessness and the inclusion of homelessness in their curriculum. The aim of the qualitative arm was to explore student perceptions and preferences about the relevance of learning about the homeless, as part of curriculum addressing health equity and care delivery to vulnerable patients. We chose the mixed methods approach to provide depth and reasoning behind student attitudes and perceptions, in part because we hypothesized that student attitudes would be strongly positive toward the homeless as reflected in the existing literature [10,13,20].The study was approved by the University of Southern California Office for the Protection of Human Subjects.

#### Survey

The survey was distributed to students by email using Qualtrics© during the first week of school prior to exposure to any curriculum. Students were given two weeks to respond with one reminder. Students responses were confidential, and students were assured that responses would be anonymous to faculty.

The survey consisted of 32 questions in total. Six questions asked about student demographics [age, gender, race/ethnicity, geographic setting in which they spent most of their life (rural, suburban, urban), personal history of homelessness, and prior experience serving the homeless in the past 5 years (in hour categories)].

The main dependent measure was the Health Professionals’ Attitudes Toward the Homeless Inventory (HPATHI), a 19-item instrument (see Table 1) with good internal consistency reliability (Cronbach’s alpha of 0.88), test-retest reliability (Pearson r = 0.69) and good concurrent validity that assesses attitudes of health care professionals towards people experiencing homelessness [21,22]. We chose the HPATHI over the ATHI (Attitudes Toward the Homeless Inventory) [23] because the HPATHI contains questions specific to the role of the healthcare provider or healthcare system in the care of the homeless. Attitudes are determined by mean scores (with higher scores representing more positive attitudes) for all 19 questions and for three sub-scales: personal advocacy (9 questions), social advocacy (6 questions) and cynicism (4 questions; three of which are negatively worded and reverse-coded). Respondents agree or disagree with statements using a 5-item, Likert-type scale. Overall and sub-scale scores range from 1 to 5, with 5 indicating strong agreement.

**Table 1.** Health Professionals’ Attitudes Toward the Homeless Inventory (HPATHI) and Learning Attitudes Scale Results for Individual Questions, University of Southern California, 2019.

Question	Sub-scale	Mean Score (SE)
**HPATHI** (19 questions)		
Homeless people are victims of circumstance	Social advocacy	3.72 (0.11)
Homeless people have the right to basic health care	Social advocacy	4.63 (0.08)
Homelessness is a major problem in our society	Social advocacy	4.79 (0.07)
Homeless people choose to be homeless	Cynicism	3.73 (0.10)
Homeless people are lazy	Cynicism	3.81 (0.10)
Health-care dollars should go toward serving the poor/homeless	Social advocacy	4.00 (0.11)
Comfortable being PCP for homeless person w/major mental illness	Social advocacy	3.82 (0.13)
Comfortable being part of a team providing care to the homeless	Social advocacy	4.51 (0.07)
Comfortable providing care to different minority/cultural groups	Cynicism	4.77 (0.06)
Overwhelmed by the complexity of problems of homeless people	Cynicism	2.81 (0.16)
Understand patient priorities more important than my medical recs	Personal advocacy	3.89 (0.11)
Providers should address physical/social problems of homeless	Personal advocacy	4.33 (0.09)
Entered medicine because I want to help those in need	Personal advocacy	4.75 (0.06)
Interested in working with the underserved	Personal advocacy	4.68 (0.06)
Enjoy addressing psychosocial issues with patients	Personal advocacy	4.00 (0.12)
Resent the time it takes to see homeless patients	Personal advocacy	4.05 (0.12)
Enjoy learning about the lives of my homeless patients	Personal advocacy	4.24 (0.08)
Social justice is an important part of health care	Personal advocacy	4.49 (0.10)
Caring for homeless is not financially viable for career	Personal advocacy	3.69 (0.11)
**Learning Attitudes Scale** (7 questions)		
PA students should be exposed to teaching about the homeless		4.58 (0.08)
Would like clinical experiences involving the homeless		4.37 (0.09)
Would like training to include encounters with unsheltered homeless		4.28 (0.09)
Learning about the homeless will help me care for other patients		4.53 (0.08)
PA students should be taught about health disparities and inequities		4.23 (0.10)
Learning about homelessness helps learn about health disparities/inequities		4.72 (0.08)
Comfortable being PCP for homeless person w/major mental illness		4.60 (0.07)

SE = Standard Error

A further seven questions (using the same 5-point Likert scale) were designed to create a scale of ‘Learning Attitudes’ (Table 1). Questions asked about the value and relevance of, preferences for and comfort with learning about and working with patients who experience homelessness. These questions were developed from the existing literature [21–23], revised by the research team (CF, GS, EL and DL) to meet the study aims, and then pre-tested with second- and third-year students and further refined.

Analyses of the survey data were conducted using STATA 13.1 [24]. First, we affirmed that the seven items measured by the Learning Attitudes scale could reasonably be combined into one scale using Cronbach’s alpha. We then calculated frequencies of the study demographics, mean values for the HPATHI total score and subscales, and examined bivariate relationships between the study demographics and HPATHI scores (Table 2). Statistical significance was set at a p-value of <0.05. We tested for differences in the HPATHI scores across groups for significance using two-way ANOVA. Demographic variables with response options that contained fewer than 5 respondents were combined with other categories to assure at least 5 respondents.

**Table 2.** Health Professionals’ Attitudes Toward the Homeless Inventory (HPATHI) and Learning Attitudes Scale Score Results by Student Demographics by Mean Score (Standard Error), University of Southern California, 2019.

	N (%)	HPATHI (n = 59)	Personal Adv (n = 59)	Social Adv (n = 59)	Cynicism (n = 59)	Learn Att. (n = 60)
**Cohort Total**	60	3.97 (0.04)	3.85 (0.04)	4.26 (0.05)	3.02 (0.06)	4.47 (0.07)
**Age (years)**						
≤25	23 (38.3)	3.89 (0.06)	3.76 (0.06)	4.14 (0.09)	3.13 (0.08)	4.27 (0.12) *
26–28	21 (35.0)	4.04 (0.04)	3.93 (0.05)	4.35 (0.08)	3.04 (0.11)	4.59 (0.09) *
29+	15 (25.0)	4.05 (0.08)	3.92 (0.08)	4.37 (0.10)	2.79 (0.09)	4.66 (0.12) *
**Gender**						
Female	49 (81.7)	3.97 (0.04)	3.99 (0.08)	4.44 (0.14)	2.92 (0.13)	4.56 (0.14)
Male	10 (16.7)	4.05 (0.09)	3.84 (0.04)	4.24 (0.06)	3.03 (0.07)	4.46 (0.08)
**Race/ethnicity**						
Asian	19 (31.7)	4.01 (0.05)	3.94 (0.05)	4.21 (0.08)	2.91 (0.09)	4.65 (0.08)
Hispanic	17 (28.3)	3.97 (0.06)	3.83 (0.08)	4.35 (0.10)	2.98 (0.13)	4.51 (0.10)
White	22 (36.7)	3.93 (0.07)	3.81 (0.07)	4.23 (0.09)	3.14 (0.09)	4.28 (0.14)
**Experience with homelessness**						
No	47 (78.3)	3.96 (0.04)	3.85 (0.04)	4.22 (0.06)	3.03 (0.07)	4.44 (0.08)
Yes	13 (22.0)	4.00 (0.08)	3.88 (0.09)	4.39 (0.13)	3.00 (0.11)	4.60 (0.13)
**Served homeless past 5 yrs**						
Never	7 (11.7)	3.79 (0.16) *	3.98 (0.15) *	3.63 (0.17) *	3.14 (0.14)	4.29 (0.30)
1–20 hours	15 (25.0)	3.96 (0.05) *	4.14 (0.08) *	3.88 (0.06) *	2.93 (0.08)	4.32 (0.11)
21–60 hours	18 (30.0)	4.13 (0.05) *	4.48 (0.08) *	3.97 (0.07) *	3.13 (0.13)	4.69 (0.10)
61+ hours	20 (33.3)	3.89 (0.06) *	4.23 (0.11) *	3.81 (0.05) *	2.95 (0.09)	4.46 (0.12)
**Geographic area** [3]						
Rural	6 (10.0)	3.84 (0.21)	3.70 (0.18)	4.14 (0.28)	3.04 (0.15)	4.00 (0.37) *
Suburban	30 (50.0)	3.99 (0.04)	3.87 (0.04)	4.32 (0.06)	3.03 (0.09)	4.62 (0.07) *
Urban	23 (38.3)	4.01 (0.06)	3.89 (0.06)	4.24 (0.08)	2.98 (0.09)	4.42 (0.10) *

SE = Standard Error, *p = <0.05

Legend: Personal Adv = Personal Advocacy, Social Adv = Social Advocacy, Learning Att = Learning Attitudes Scale

#### Focus groups

FGs take advantage of group dynamics by stimulating conversation [25]. The process helps participants identify, reflect on, and clarify their own views. Qualitative methods, such as FGs, are useful for issues involving differing opinions, values and perceptions, especially among groups, such as students, that do not exercise power [25]. We selected this method for its ability to elicit both individual and group responses. We used the literature [12–14,20,25–27,28–30] as a basis for semi-structured, open-ended questions and probes addressing our research question. The question guide (Table 3) was designed to establish student perceptions about the need to prepare to care for vulnerable populations before progressing to explore their desire or preferences for including homeless patients in the required curriculum. Our research team comprised diverse expertise from medical education and educational research (CF, DL), clinical practice (DL, CF), public health (GS) and anthropology (EL), designed to capture differing perspectives. Our aim was to conduct FGs to theme saturation. The FGs were held after the first six weeks of the student’s first semester, after exposure to 4 weeks of basic sciences curriculum. Participants were recruited using two group emails to the class. The FGs were conducted in two conference rooms on site by two experienced moderators [31] with no role in student assessment. Students received lunch during the FG and no compensation was offered. The FGs were audiotaped and transcribed verbatim.

**Table 3.** Focus group question guide for learning about homeless and vulnerable, University of Southern California, 2019.

Key Questions	Probes
1. What is your concept of ‘vulnerable’ patients or populations?	- In your future practice, do you expect to see vulnerable patients? - If so, what proportion of your patients do you anticipate will be considered ‘vulnerable’?
2. Tell us about the relevance of learning about the homeless to your role as a future physician assistant (PA).	- How comfortable are you about seeing homeless patients? Tell us why you are comfortable or uncomfortable. - What knowledge or skills can you gain from learning about homeless persons and their healthcare experiences? - What is your previous exposure to the homeless?
3. What types of learning experiences around homelessness would be effective for you to learn skills for your future practice?	- What foundational knowledge or preparation do you need before seeing homeless patients? - What do you think of class lectures and panels of experts including patients? - What do you think of clinical rotations on the street as a learning experience? [What benefits do you see? What concerns do you have?]
4. How might learning about the homeless experience with healthcare help you in caring for other vulnerable patients in future practice?	- How might learning about homeless patients teach you about other patients or clinical conditions? - How should programs prepare their students to care for vulnerable populations?
5. Please share any other thoughts and feelings about the value of learning about vulnerable populations and communities for your professional development.	No probes

In the data analysis, transcripts were independently read and coded by three researchers (CF, DL, and GS) who used constant comparison [32,33] to interpret the data and develop his/her own major themes and subthemes. The coders then met to extract major themes and subthemes. A fourth coder (EL) acted as adjudicator for potential disagreements.

## Results

### Survey

The response rate was 100% (60 of 60). One student provided incomplete responses and was not included in the analysis. Respondents (Table 2) were mostly female (83.0%) and diverse (with 32.2% self-reporting Asian race/ethnicity and 28.8% Hispanic/Latino). One-fifth (22.0%) had personal experience with (or knew someone close to them who had experienced) homelessness. Most (88.1%) had served the homeless in the past 5 years. A small proportion had spent most of their life in a rural setting (10.2%).

The overall HPATHI mean score was 3.97 out of 5, indicating positive attitudes towards people who are homeless (Table 1). The highest mean subscale score (4.26) was for the social advocacy sub-scale (e.g., ‘homeless people have the right to basic health care’; ‘health care dollars should go toward serving the poor/homeless’). Lower mean scores were seen for the personal advocacy (3.85) (e.g., ‘providers should address physical/social problems of the homeless’) and cynicism sub-scales (3.02) (e.g., ‘homeless people are lazy’, reverse-coded so that a higher score is less cynical) See Table 2 for HPATHI subscale scores.

The seven items of the Learning Attitude scale (Table 1) had a Cronbach’s alpha of 0.89 (above the recommended cut-off of 0.80) [34], indicating adequate internal consistency reliability. The mean Learning Attitude scale score was 4.47 out of 5 indicating highly positive views about the inclusion of homelessness as a curricular exposure (e.g. ‘learning about the homeless will help me care for other patients’, ‘learning about homelessness helps me learn about disparities/inequities’).

There were differences in mean HPATHI and learning attitude scale scores by student demographics (Table 2). Students who reported serving the homeless in the past 5 years had higher (more positive) HPATHI mean scores and personal and social advocacy subscale scores. Older students had a more positive (higher) HPATHI mean score (4.27 among those ≤25 years, 4.59 for ages 26–28, and 4.66 for 29+ years, p < 0.05). Rural background (6 students) was significantly associated with a lower Learning Attitude score (4.00 vs. 4.42 for urban geography and 4.62 for suburban, p < 0.05).

### Focus groups

The class email invitation sent twice resulted in 16 volunteer students who participated in three FGs (A, B and C) on the same day, with 8, 4 and 4 students respectively. All participants were female with age range of 22 to 45 years. Theme saturation was achieved by the third FG in that no new major themes emerged. After independent coding, the 3 coders met and agreed on four major themes. Within each major theme the coders and the adjudicator agreed on a range of 3 to 4 subthemes supported by typical quotes (Table 4). Students showed overall agreement on most areas apart from the potential of requirement for a Street Medicine rotation. The major themes are presented below.

**Table 4.** Focus group themes, subthemes and typical quotes, University of Southern California, 2019.

Major themes	Subthemes	Typical quotes
1. Vulnerable patients cannot advocate for themselves	Navigating the health system	‘ … patients who don’t know how to advocate for themselves … (because of) lack of awareness of the system … or English language skills or cognitive deficits … ’ FG A, Student G‘ … a vulnerable patient isn’t able to navigate or advocate for themselves when they needed something’ FG B, Student B
	Social determinants of health	‘ … related to the migrant population that have barriers to the social determinants of health, that is, education, low socio-economic status, the neighborhood … those marginalized or in underserved communities.’ FG C, Student W ‘ … not just not having the ability to go see a provider, but … you can’t really follow or be compliant because of not having the financial needs or maybe the emotional ability to complete what your provider would want.’ FG B, Student A
	Experience of discrimination and bias	‘ … minority groups that have historically faced discrimination or abuse within the healthcare system and might not have trust in the current medical system.’ FG A, Student A
2. Learning about homelessness is relevant to future practice	Increases empathy for other vulnerable populations	‘ … the relevance for me with learning about the homeless population is that it forces you to bring human side of medicine back … and makes you realize why you got into this profession and makes it more meaningful and personal.’ FG C, Student X ‘ … I’d like to hear what they’ve had to do to get through day by day, where they were and where they are now, where some of the emotions, some of the fear … because that would give life to what we’re trying to imagine how it would be.’ FG C, Student Y
	Learn to manage medical and social complexity	‘ … how to best triage what is the most important thing to take care of and what can we get to later. That would be a good skill overall ‘we can learn ‘working with homeless communities.’ FG A, Student A ‘ … learning how to navigate medications or follow ups when they don’t have the resource to get there will help (me) learn how to be a better provider for vulnerable people.’ FG B, Student B ‘ … learning more about different ways to overcome barriers such a transportation and follow-up appointments would be pretty beneficial for us.’ FG A, Student B
	Improves cross-cultural and overall communication skills	‘ … patient preferences, patient autonomy, and getting to know the patient and who they are as an individual … I think it will be different and more fruitful (working with the homeless) if we hear it in their words to inform how can we approach a situation sensitively in a culturally competent manner.’ FG C, Student W
	Homelessness is a current societal and public health issue.	‘I think understanding why major US cities are going through a housing crisis will help us increase our empathy.’ FG A, Student D ‘ … the relevance of learning about the homeless is that it’s not just my problem or their problem, it’s all of our problem.’ FG C, Student Y
3. Prefer multiple methods of learning about the homeless	Prefer cased-based learning within the curriculum	‘(I would like to hear about) faculty’s own cases – people they have followed over time so we see what they’ve been doing every couple weeks or so.’ FG B, Student D‘ … lectures or panels with actual patients or individuals that experienced (homelessness) would be a better way of bringing life to the material … hearing from their perspective.’ FG C, Student W
	Need for foundational tools and skills to care for the homeless	‘ … before we address the complexities of treating or helping all these patients, we need a strong basic foundation.’ FG A, Student F ‘I (suggest) adding on more lectures about cultural competency and in general about what the vulnerable population might be experiencing.’ FG C, Student W
	Experiential learning	‘ … having a PA work with the staff physician at street medicine clinic and mobile clinic would be an incredible (exposure), something for us to look to, they would be a great example for us in our future (practice).’ FG B, Student E ‘ … I would like to shadow out in the field. That would be really helpful, to bridge that gap between being in class and actually doing.’ FG C, Student Z
4. Anticipated fear and anxiety about patient care in a street setting	Concern about physical safety	‘I’m hesitant to say that (street medicine) should be a required rotation because of the personal safety issue and a lack of comfort providing care in that particular (context) versus providing care in a traditional (clinic) setting.’ FG B, Student E‘I don’t think (street medicine) should be a required clinical rotation. (Students) should be able to choose that as (an alternative to) the second family medicine rotation.’ FG B, Student B‘ … safety has to be addressed … when you’re going into skid row where a lot of homeless populations are, … you’re not in a controlled setting.’ FG C, Student X
	Psychological distress/self-care/burnout	‘ … make mental health services available for students on a street medicine rotation, or some type of debriefing frequently with either a mental health professional or a preceptor and the student so that emotional issues … are addressed promptly.’ FG B, Student E‘ … I expect to be emotionally prepared for the population. I wouldn’t expect to be just thrown out being able to deal with everything coming my way.’ FG B, Student A
	Mixed reaction to required street medicine rotation	‘I would say (the street medicine rotation should) not be required because while I do love that our program is primary care and street medicine-focused, some of our peers are not here for that aspect of the profession.’ FG A, Student B‘ … it depends on the cost of making it required and what other rotation you’re going to (substitute it for).’ FG A, Student C‘ … a required street medicine rotation means that (practitioners) who do not have that experience on the streets will be able to take what we gain and … apply it in clinic. And when we see a homeless patient (in clinic) we would also be resourceful … ’ FG C, Student W

## Vulnerable patients cannot advocate for themselves

Within all three FGs, students demonstrated strong agreement that vulnerable patients comprised those who could not advocate for themselves and, even when they could access healthcare services, may have challenges adhering to or complying with recommendations from providers. They agreed that not only did vulnerable patients not know how to navigate health systems they also could not identify advocates to assist them and hence suffered multiple disadvantages on health outcomes. Students associated these limitations with a history of experiences of discrimination or bias, social determinants of health such as education and environment, communication difficulties associated with language barriers, and limited health literacy or cognitive deficits. Students provided specific examples of vulnerable patients based on their own prior exposures and experiences, and listed among them, immigrants, those with limited English proficiency, refugees, the elderly, and victims of violence. None of the students listed homelessness as a condition associated with vulnerability, and a student commented on this apparent oversight:

I think it is interesting we didn’t list homelessness as a vulnerable population; but I think we were thinking about the nitty gritty of what a vulnerable person was. A homeless person is so obviously a vulnerable person that we didn’t even think to point them out. FG B, Student B

Students also personalized their own experiences with vulnerable patients based on their past exposure and intended future practice. Students agreed that they will all see vulnerable patients in their future practice, no matter their chosen specialty, and that the proportion of vulnerable patients would depend on the practice context and setting. For example, one student said:

I think it has a lot to do with our lived experiences and our biases that we carry with us, so specifically for me, it would be related to the migrant population. Individuals that have barriers to the social determinants of health, to accessing care … whether that is education, low socio-economic status, or the neighborhood they are surrounded by. FG C, Student W

Some students considered it within the scope of their future practice to advocate for the vulnerable. For example, one student commented:

Particularly my goal would be to work in a lesbian, gay, transsexual clinic for queer patients … this group is more likely to experience discrimination and be more disenfranchised …. FG A, Student A

## Learning about homelessness is relevant to future practice

When responding to questions or probes about the relevance of working with or learning about homelessness, students agreed that they were open to this topic in their curriculum. Some went further by remarking that the homeless represented multiple aspects of vulnerability and by learning about their circumstances in healthcare students would be better equipped to care for all vulnerable groups in their future practice. One student remarked:

a relevant medical curriculum … will include learning about current day issues and homelessness is an issue (of) many cities with housing prices rising. It fits that we are learning about this because we will all have some interaction with unsheltered or homeless individuals. FG A, Student A

Students provided specific reasons why working with the homeless would give them skills to care for the vulnerable. They listed the following categories of learning (see subthemes, Table 3): learning to have greater empathy and to ‘walk in their shoes’; learning to manage complexity both in the medical and the social context; improving cross-cultural communication skills; and integrating an understanding of the homeless condition as a universal problem faced by society. Areas of specific learning that they identified included mental health, substance abuse, depression and anxiety, managing medications and triaging in the real world. Comments about the direct relevance of caring for the homeless to caring for vulnerable patients in general included:

… (this experience to learn about the homeless) brings humility and dignity to the care that we provide … to not further isolate them but to treat them on a more personal level … and (also) other individuals that need our help. FG C, Student W

## Preference for multiple methods of learning about the homeless

Students called for a firm foundation of tools and skills to care for the homeless citing the ubiquitous use of case-based learning and experiential learning opportunities. Case-based learning was consistently given as a preferred learning strategy in all FGs. Students commented that hearing about experiences ‘from the field’ made them more eager to learn. One student said:

… case studies are really important … they make things stick and they get me excited for what’s to come. FG A, Student A

When asked how working directly with the homeless would impact their future practices, students also stated that experiential learning would allow them to work with a variety of health care professionals and expose them to other scopes of practice. The desire to bridge the gap between the classroom and actual practice as well as practice gaps between professions underpinned this sentiment. One student commented:

… a street medicine curriculum really reminds us how broad our scope of care can be; how broad the scope other interdisciplinary or inter-health professionals can be in terms of the unique challenges that present in (caring for) those who are experiencing homelessness. FG B, Student E

## Anticipated fear and anxiety about patient care in a street setting

Student responses varied when discussing whether a street medicine clinical rotation should be required. Opinions were associated with specific concerns. A common subtheme was anticipated fear and anxiety about patient care in a street setting. Students who were eager for a street medicine rotation made such comments as:

I would love the opportunity to do something like that, like shadowing … you’re ready to start practicing and you really get out there and see what it’s like and get practice in the field. FG C, Student X

… it would be really nice if we had more reinforcement with real-life experience by making street medicine a required rotation and not just an elective. FG A, Student E

Other students conveyed concerns about physical safety, psychological distress, self-care, and provider burnout. Physical safety generated the most concern and was the main reason students felt that a street medicine rotation should not be required. One student voiced the following:

… I agree about pushing someone out of their comfort zone as that is where they most need to grow, but safety and resources are not guaranteed on the streets, so making a required clinical rotation in street medicine is asking too much of a personal sacrifice. FG B, Student B

## Discussion

We conducted a mixed-methods study to explore matriculating students’ attitudes related to learning about and working with people experiencing homelessness. While there are descriptions of curricula addressing the homeless [9,10,14,27,35], no prior study has examined the integration of a curriculum inclusive of the unsheltered homeless as part of teaching about disparities and health equity. Furthermore, most curricula described are optional or voluntary and involve students seeing patients in shelters rather than on the street. Our study provides a unique first step in the form of a needs assessment. We affirmed the findings of previous studies [9,10,13,14,35,36–38] showing overall student empathy for and positive attitudes toward helping and advocating for the homeless. We found that prior experience with the homeless [4] was associated with higher mean HPATHI and subscale scores, underscoring the role of immersion and exposure in building empathy. Only one prior study [12] has reported no change in student attitudes with curricular exposure; however this study did not expose students to an intentional curriculum or experiential learning interventionOur Learning Attitudes survey provides new information demonstrating highly positive student attitudes about the inclusion of homelessness healthcare in the required curriculum. However, our results indicate a dichotomy with students from rural backgrounds having a statistically significantly lower Learning Attitude score. We are not able to draw conclusions from this association as the number of students (6) from rural backgrounds was low.

Our FGs affirmed matriculating students’ desire to work with vulnerable populations in their future practice. Students appreciated that caring for the homeless is an appropriate proxy for learning about other vulnerable populations [8]. Strong didactic foundational components and case-based learning was clearly their preferred learning format during the pre-clinical phase followed by clinical exposure. The FGs also revealed hitherto unreported mixed reactions about and emotions associated with working in a street setting. Specifically, students recognized the significant mental, physical and social stresses associated with working with such a disadvantaged group and advocated for mental health preparedness.

Our study suggests that students find focusing on a single, complex vulnerable population to learn transferable knowledge and skills acceptable. While we addressed the homeless population, educators may also consider other vulnerable groups (such as refugees or the incarcerated) to teach similar skills. Our students view the homeless as an important but not the only vulnerable group, stressing the importance of exposure to other vulnerable groups. We recommend that educators consider giving students choices when designing rotations in underserved settings, and specifically, inquire about their concerns about the location of such rotations. Educators should also offer a robust longitudinal wellness and self-care curriculum to prepare students. This may be an important early approach to insulating future providers against burnout and ’ compassion fatigue’ [39–41].

Strengths of our study include the use of both quantitative and qualitative methods, the 100% response rate to the survey, reaching theme saturation and the diversity of opinions. Limitations include that this is a single-institution study involving one class of students at a PA program that emphasizes service to the underserved community in its mission. Students open to working with the homeless may have self-selected into our program. While not all health professions involve students in clinical rotations, we believe our findings are relevant to any curriculum that addresses homelessness and health disparities through didactic teaching and clinical experiences with underserved patients.

In summary, we conducted a needs assessment for a longitudinal, health equity curriculum inclusive of people experiencing homelessness. Next steps include integration of study findings into the new equity-based curriculum; assessment of its effectiveness; and an exploration of the role of location (clinic, hospital vs. street) on student learning. Student concerns about a nontraditional practice setting will be addressed to best prepare them for rotations where physical or psychological safety are issues (e.g. clinics located in high crime areas and corrections medicine). Our study helps inform other health professions educators as clinical training moves beyond hospitals into novel community settings where healthcare is most needed, with the promise of new and effective teaching opportunities for our learners.
